# Early diagnosis and treatment referral of children born small for gestational age without catch-up growth are critical for optimal growth outcomes

**DOI:** 10.1186/1687-9856-2012-11

**Published:** 2012-05-04

**Authors:** Christopher P Houk, Peter A Lee

**Affiliations:** 1 Medical College of Georgia, 1120 15th Street, Room BG1007, Augusta, GA, 30912, USA; 2 Penn State College of Medicine, Milton S. Hershey Medical Center, PO Box 850, Hershey, PA, 17033-0850, USA; 3 Indiana University School of Medicine, The Riley Hospital for Children, 702 Barnhill Dr, Indianapolis, IN, 46202, USA

**Keywords:** Hormone, GH therapy, Referral age, Short-statured, Optimal height acceleration, Gestational age, Prepubertal height, Adult height

## Abstract

Approximately 10% of children born small for their gestational age (SGA) fail to show catch-up growth and may remain short-statured as adults. Despite treatment guidelines for children born SGA that recommend referral for growth hormone (GH) therapy evaluation and initiation by ages 2 to 4 years, the average age of GH treatment initiation is typically much later, at ages 7 to 9 years. Delayed referral for GH treatment is problematic as studies show younger age at GH treatment initiation in children born SGA is an independent predictor for responses such as optimal growth acceleration, normalization of prepubertal height, and most importantly, adult height (AH). This review discusses the importance and associated challenges of early diagnosis of children born SGA who fail to show catch-up growth, contrasts the recommended age of referral for these patients and the average age of GH treatment initiation, and discusses studies showing the significant positive effects of early referral and treatment with GH on AHs in short-statured children born SGA. To optimize the eventual height in short-statured SGA children who fail to manifest catch-up growth, a lowering of the average age of referral for GH therapy evaluation is needed to better align with consensus recommendations for SGA management. The importance of increasing parental and physician awareness that most children born SGA will do well developmentally and will optimally benefit from early initiation of GH treatment when short-statured is addressed, as is the need to shift the age of referral to better align with consensus recommendations.

## Introduction

Being small for gestational age (SGA) at birth has many causes, including fetal, placental, maternal, and environmental factors [1,2]. SGA is typically diagnosed when birth weight and/or length are at least 2 standard deviations (SDs) below the mean for gestational age, using appropriate reference data [1,3]. Children born SGA comprise a heterogeneous group with a broad spectrum of clinical characteristics [4]. SGA may occur alongside intrauterine growth retardation (IUGR) and/or premature birth, or may be diagnosed at term in the absence of any prenatal complications. The etiology of SGA is frequently unknown, and current estimates suggest that 40% of SGA births will have no identifiable pathology. Of those 60% of SGA infants where an etiology is identified, about 50% involve maternal factors, 5% involve fetal abnormalities, and less than 5% are felt to be due to placental pathology. Maternal factors associated with SGA include inadequate nutrition; hypoxia; diabetes mellitus; drug use and abuse; vascular, hematologic, and renal disorders; infection; and sociodemographic factors. Fetal causes include congenital anomalies; chromosomal abnormalities; infection; and hormone abnormalities involving insulin, leptin, thyroid hormones, and insulin-like growth factors (IGF-1 and IGF-2). Placental factors that may result in SGA include placental insufficiency, infarction, abruption, and vascular abnormalities [1,5-8].

The available SGA incidence and prevalence data are limited due to insufficient or inconsistent records for birth length and gestational age in national databases [9]. A population-based study including 3650 healthy full-term neonates born in Sweden over a 3-year period found 5.4% (N = 198) were diagnosed SGA, defined as <−2 SDs in birth length and/or weight [10]. Within the group of SGA children, 1.5% were both underweight and short, 2.4% were short only, 1.6% were underweight only. The estimated incidence of SGA births, using the definition of <−2 SDs in length or weight (equivalent to the 2.3 percentile), is 1 in 43, making SGA incidence relatively high compared with other growth disorders [11]. Most children born SGA show catch-up growth, generally defined as growth velocity (cm/year) greater than the median for chronologic age and gender, within the first 2 years of life; however, approximately 10% fail to show catch-up growth and may remain short-statured as adults [1]. Growth hormone (GH) therapy has been approved for long-term therapy of growth failure in short-statured children born SGA who show no evidence of catch-up growth by age 2 to 4 years [1,3,12]. The mechanisms underlying catch-up growth remain unclear [13]. Lack of catch-up growth has not been associated with any specific SGA etiology; however, preterm birth with less than 32 weeks gestation has been associated with a greater risk for no catch-up growth [3,14].

The average age of GH treatment initiation in short-statured children born SGA is typically many years later than the recommended age of 2 to 4 years [3,15-17]. This frequent delay in GH treatment is problematic as older age at GH therapy initiation is associated with significantly reduced growth response [15,18-20]. This review discusses the importance and associated challenges of early diagnosis of SGA with failure of spontaneous catch-up growth. We contrast the recommended age of referral and GH treatment initiation, according to consensus-based guidelines, with the average age(s) reported in treatment studies; we then review the significant positive benefits of early GH treatment on optimal height outcomes in short-statured patients born SGA.

### Diagnosis of SGA

Accurate gestational age dating and measurement of birth weight and length are critical for SGA diagnosis [3]. There are several challenges to achieving accurate gestational age dating and accurate birth weight and length measurement. The accuracy of gestational age dating depends on the method used. An estimate based on last menstrual period produces greater error compared with clinical or obstetric estimates using early ultrasound assessment [21-24]. Accurate assessment of growth restriction requires careful and precise neonatal measurements, which are essential to establish size relative to gestational age [25-27]. Additionally, appropriate use of population-relevant reference growth curves is also necessary for accurate diagnosis of SGA [28,29].

Discriminating between pathologic and constitutional SGA is difficult, and guidelines for the selection of appropriate reference comparison data are evolving. The anthropometric definition of SGA does not account for background growth-modifying factors, such as maternal height, weight, ethnicity, and parity [3,30]. These modifying factors can be used to statistically model a corrected birth weight and/or length, and may increase the likelihood of identifying abnormal fetal growth compared with constitutional smallness [30]. This approach to growth assessment adjusts for physiological variation, calculates true growth potential, and creates individually customized fetal, neonatal, and child growth curves and birth weight percentiles [30-33]. Methodology for customized growth assessment is currently being developed and is not yet widely available.

To expedite appropriate early referral to GH treatment, early diagnosis of SGA and recognition of failure of spontaneous catch-up growth are critical [1,3]. Measurements of length, weight, and head circumference should be taken every 3 months in the first year of life and every 6 months thereafter [3]. Diminished head growth, particularly when it occurs both in utero and postnatally, is especially important to follow. Little or no catch-up head growth is a significant risk factor for poor outcome as it has been associated with widespread cognitive impairments [34]. Spontaneous catch-up growth typically occurs by age 2, and is most pronounced in the first 6 to 12 months after birth [1,3]. A child without spontaneous catch-up growth by age 3, or by age 4 in preterm infants, is unlikely to experience it later without therapeutic intervention [1,3]. It has been shown that children born SGA have a 7-fold higher risk of being short at age 18 than do children not born SGA, and children born SGA comprise 22% of adults whose height is below −2 SD scores (SDs) [35].

### Indication for GH treatment

GH treatment of short children born SGA has been approved by the US Food and Drug Administration (FDA) since 2001 and the European Medicines Agency (EMA) since 2003 [1,3,11]. Prior to initiating GH treatment, other causes of short stature must be excluded, including growth-inhibiting medication, chronic diseases, endocrine disorders, emotional deprivation, or syndromes associated with short stature. GH stimulation testing or the presence of GH deficiency (GHD) are not required prior to GH treatment of children born SGA as the growth benefit from GH treatment occurs whether GHD is present or not [36-38]. No correlation has been found between spontaneous 24-hour GH profiles or maximal stimulated GH secretion before the start of GH treatment and adult height (AH) SDS or gain in height SDS in children born SGA [37].

Initiation of GH treatment is appropriate when the opportunity for spontaneous catch-up growth has passed [1,3,35,39]. In the US, treatment is indicated in children born SGA who fail to achieve normal growth velocity by age 2. The EMA indication approves GH treatment beginning at age 4 [3]. The International Societies of Pediatric Endocrinology and the Growth Hormone Research Society consensus statement proposes that children born SGA with height below −2.5 SDs at age 2, or with height below −2 SDs at age 4, should be GH treatment eligible [3]. Thus, by age 4, initiation of GH treatment is broadly recommended in SGA children with short stature when the family feels AH is important.

### Early referral for GH treatment

The average age of GH treatment initiation in children born SGA is frequently much older than the 2- to 4-year-old age range recommended by consensus guidelines [3,15,40]. Among 360 GH-naïve, born-SGA pediatric patients participating in the American Norditropin Studies: Web-Enabled Research (The ANSWER Program®) registry, the mean age at treatment initiation was 8.4 years [15], and among 1909 children born SGA enrolled in the Pfizer International Growth Database (KIGS; a pharmacoepidemiological survey of children treated with GH) the mean age at start of GH therapy was 9.1 years (range, 3.9-13.3) [17]. Significant variation in age of referral and GH treatment initiation for short-statured SGA patients has been shown among different countries, ranging from a mean age of 6.7 to a mean age of 9.3 [16]. The older-age treatment referral and initiation is problematic as SGA patients beginning GH therapy at ages 9–10 experience lower growth velocity and have shorter AHs compared with those treated earlier [1].

The reasons for the current practice of delayed referral are unclear. One possibility is that parent attitudes and preferences regarding treatment influence physician decision making. For example, physicians were 40% more likely to recommend GH therapy for a child with short stature, not specific to SGA, if the family strongly desired GH treatment compared with a family neutral about treatment [41]. Parental realization of the importance of optimal growth may be delayed due to the presence of other comorbid conditions during the early developmental period of their child born SGA. Parents of children born with extreme SGA, comorbid systemic diseases, and high risk of mortality may feel fortunate for the survival of their child and, comparatively, may not believe short stature is as important an outcome. In particular, among children born SGA with a poor cognitive developmental prognosis, height may be considered unimportant. Parents may simply be pleased to have a reasonably healthy child after a difficult beginning and they may not seek treatment for short stature until the child is older than the optimal referral age. Parents need to be educated that most children born SGA will do well developmentally, that children without spontaneous catch-up growth are highly likely to remain short as adults, and that GH treatment, when initiated at an early age, will improve childhood growth rate and AH.

Alternatively, delayed referral could be due to beliefs of the treating pediatrician or primary care physician. Children born SGA typically leave the care of neonatologists before they are 2 years old and receive care from a pediatrician or primary care physician. Pediatricians and primary care physicians may not consider height to be a concern among patients who are short but healthy until the optimal referral age has passed. It is important to educate families and physicians about the significance of the age of referral because studies show younger age at GH treatment initiation is an important predictor of response to GH therapy [15,18,19].

### Younger age and growth response to GH treatment

Effectiveness of GH treatment in short-statured children born SGA has been well demonstrated [12,13,42,43]. A 4-year study of GH treatment in SGA and GHD children enrolled in the NordiNet® international outcome study (IOS) demonstrated similar height improvement in SGA and GHD children, supporting the idea that GH treatment in non-GHD patients is as effective as it is in GHD children [44]. The cumulative mean height standard deviation score (∆HSDS) was 1.60 in SGA and 1.55 (*P* = 0.412) in GHD children, and height was within the normal range after 4 years of GH treatment in 68% of SGA children and 79% of GHD children.

A 2-year study of GH treatment in children enrolled in the ANSWER Program registry found the largest ∆HSDS at 1 and 2 years occurred in patients with multiple pituitary hormone deficiency (MPHD) (0.85, 1.20; year 1, year 2) and SGA (0.80, 1.18), compared with GHD (0.61, 1.06), idiopathic short stature (ISS) (0.54, 0.90), and Turner Syndrome (TS) (0.50, 0.82) patients [40]. After 2 years of GH treatment among children enrolled in the IOS and ANSWER Program, greater ∆HSDS was found for patients with SGA compared with GHD (1.03 versus 0.97; *P* = 0.047).

However, therapeutic response is variable and age at GH treatment initiation is a critical factor in predicting growth outcome. Mathematical models developed to predict optimal growth following GH therapy in children born SGA show younger age at treatment initiation is a key predictor of growth response [19,45]. The most important determinants of greater first-year growth during GH therapy in short children born SGA were younger age and higher dose of GH [19,45]. The models show growth velocity during the first year of treatment, which is significantly influenced by age at treatment initiation, is the most important predictor of subsequent growth, suggesting AH outcome is indicated by the initial response to GH [19]. Greater long-term growth response observed in AH SDs in children born SGA was shown to depend on the duration of GH treatment, with younger age at treatment initiation and longer phase of treatment producing greater height increases [45].

Younger age at GH treatment initiation is associated with greater short-term height response (see Table 1[15,16,19,46,47]). A comparison among children born SGA and enrolled in NordiNet IOS from 5 European countries (N = 433) found the greatest changes in height standard deviation score (∆HSDS) occurring during the first year of GH treatment in countries where children were younger (mean age 6.7) at treatment initiation (*P* < 0.0001) [16]. Among males, the change from baseline ∆HSDS in the first year of GH treatment was significantly greater for patients who started GH treatment at a younger age (ie, younger than age 11 [∆HSDS = 0.82, N = 101], than it was for patients who were older than age 11 [∆HSDS = 0.27, N = 30], *P* < 0.0001) [15]. ∆HSDS in years 1–2 of GH treatment was greater in SGA patients enrolled in the NordiNet IOS (N = 423) who were younger at GH treatment initiation [18]. ∆HSDS following 1 year of GH treatment was significantly greater in prepubertal (∆HSDS = 0.75, N = 24) compared with pubertal (∆HSDS = 0.40, N = 15, *P* = 0.016) short-statured SGA children [47]. Among very young (ages 2–5) SGA children, the greatest gain in growth velocity during the first 2 years of GH treatment occurred in those younger than age 4 (1.7 SDS, N = 16) compared with children older than 4 (1.2 SDS, N = 23, *P* < 0.05) [46].

**Table 1:** Age at treatment initiation and short-term GH treatment outcomes in children born SGA

**Study**	**N**	**Design/Duration**	**Age**^**a**^**/Model**	**Outcome (∆HSDS or statistical model)**	***P*Value**
Argente 2007 [46]	39^b^	MC, C, R, O/2 years	2 to <4 years	1.7 at 1 year; approximately 2.5 at 2 years	<0.05
			4 to 5 years	1.2 at 1 year; approximately 1.8 at 2 years	
Carvalho-Furtado 2009 [47]	39	Ob/1 year	Prepubertal	0.75	=0.016
			Pubertal	0.40	
Lee 2008 [16]	433	MC, NordiNet IOS/≥1 year	Mean age 6.7 to 9.3	6.7 years = 1.0 and 0.8; 7.6 years = 0.72; 8.3 years = 0.61; 9.3 years = 0.57	<0.0001^c^
Ranke 2003 [19]	613 year 1;	KIGS, clinical trials/2 years	Mean age 6.6/statistical models predicting growth response	Year 1: GH dose (35% of variability), age at treatment start (11% of variability)	<0.0001 <0.0001
	385 year 2			Year 2: HV in year 1 of treatment (29% of variability), age at treatment start (3% of variability), GH dose (2% of variability)	
Ross 2010 [15]	208 year 1;119 year 2	ANSWER Program registry/2 years	Mean age 8.4/males <11 years vs ≥11 years; females <10 years vs ≥10 years	Year 1 boys: <11 years = 0.82; ≥11 years = 0.27Year 1 girls: <10 years = 0.58; ≥10 years = 0.41Year 2 boys: <11 years = 1.23; ≥11 years = 0.59Year 2 girls: <10 years = 1.00; ≥10 years = 0.87	<0.0001 = 0.093 = 0.0005 = 0.56

^a^Age at treatment initiation; ^b^Data are reported for study group 1 with 2 years GH treatment; ^c^Multivariate analysis showed ∆HSDS was dependent on age at the start of treatment. ANSWER, American Norditropin Studies: Web-enabled Research; C, controlled; GH, growth hormone; ∆HSDS, change in height SDS; HV, height velocity; IOS, international outcome study; KIGS, Pharmacia International Growth Database; MC, multicenter; O, open trial; Ob, observational; R, randomized; SDS, standard deviation score.

Younger age at initiation of GH treatment is also associated with greater long-term height response (see Table 2[20,36-38,45,48]). In a long-term prediction model of height SDS at the onset of puberty, younger age at GH therapy initiation was associated with greater height outcome at puberty in short-statured SGA children (N = 150) [20]. Younger age at GH treatment initiation was also associated with better AH following long-term GH treatment in short SGA children (N = 38) [49]. Among 77 short-statured, prepubertal children born SGA, better catch-up growth to AH in response to GH treatment was noted in children who were younger at the start of GH treatment (r = −0.56, *P* < 0.0001) [36]. Among children treated for >2 years before puberty, the mean gain in height was 1.7 SDS, compared with 0.9 SDS (*P* <0.001) when treatment was initiated <2 years before the onset of puberty [36]. Long-term, continuous GH treatment (mean duration, 7.8 years), in children born SGA (N = 54) resulted in normalization of AH in most, with 98% reaching an AH within their target height range; younger age at the start of GH treatment was significantly associated with greater gain in height SDS from the start of GH treatment until AH (r = −0.36, *P* <0.01) [37]. Alternatively, GH treatment initiation in short adolescents born SGA (mean age, 12.7 years) has produced a more limited growth response with a mean height gain of 1.1 SDS and 47% of treated patients reaching AH in the normal range for the general population [50].

**Table 2:** Age at treatment initiation and long-term GH treatment outcomes in children born SGA

**Study**	**N**	**Design/Duration**	**Age**^**a**^**/Model**	**Outcome (∆HSDS or statistical model)**	***P*Value**
Dahlgren 2005 [36]	77	Ob/prepubertal to FH	Prepubertal during >2 years GH therapy vs prepubertal during <2 years GH therapy	Mean gain FH SDS prepubertal for >2 years GH therapy = 1.7; prepubertal for <2 years = 0.9	<0.001
de Ridder 2008 [47]	150	Data from 2 previous GH trials; R, DB/5 years[38]; R, C/3 years[48]	Median age 7.5/Statistical model predicting HSDS at puberty	Age at start (−0.27 estimated coefficient)Other significant predictors: HSDS at start (0.71 estimated coefficient), target height SDS (0.13 estimated coefficient), GH dose X IGF-I SDS at start (−0.29 estimated coefficient), gender (−0.34 estimated coefficient)	<0.0001 = 0.009 to <0.0001
Ranke 2010 [45]	161	KIGS, clinical trials/7.7 years	Median age 7.8 years/Statistical models predicting AH SDS and ∆HSDS	70% of variability in adult height SDS: HSDS at GH start, ∆HSDS 1st year on GH, years on treatment [younger start, longer phase], maternal HSDS, length SDS at birth, SRS diagnosis	NR
				60% of variability in ∆HSDS: ∆HSDS 1st year GH, H-MPH SDS at GH start, years of GH treatment [younger start, longer phase]	
van Pareren 2003 [37]	54	MC, R, DB/mean 7.8 years	Mean age 8.1/∆HSDS correlations	∆HSDS from start of GH treatment to AH negatively correlated with age at treatment start: r = −0.36	<0.01

^a^Age at treatment initiation. AH, adult height; C, controlled; DB, double-blind; FH, final height; GH, growth hormone; HSDS, height SDS; ∆HSDS, change in height SDS; IGF-I, insulin-like growth factor; KIGS, Pharmacia International Growth Database; MC, multicenter; MPH, mid-parental height; NR, not reported; Ob, observational; R, randomized; SDS, standard deviation score; SRS, Silver-Russell syndrome.

In addition to better growth outcomes, the greater growth response to GH treatment among younger patients born SGA may result in cost savings. A lower GH dose of approximately 33 mcg/kg/day from treatment initiation to AH is effective in children born SGA not extremely short-statured (eg, height above −3 SD), especially if treatment begins at ages 4–6 [51]. Children beginning GH therapy in late prepuberty or extremely short-statured at treatment initiation often receive a higher dose (≥50 mcg/kg/day) for optimal short-term catch-up growth, and then can be tapered back to 33 mcg/kg/day. Thus, a better cost of GH treatment-to-height benefit outcome ratio may be achieved when GH treatment begins at an early age because a greater growth response occurs at a lower GH dose.

### Safety of GH treatment in children born SGA

The long-term safety of GH treatment in childhood has been under intense recent discussion due to the Safety and Appropriateness of Growth hormone treatments in Europe (SAGhE) mortality data from France suggesting increased all-cause, bone tumor-related, and circulatory system disease-related mortality over the mean 17.3-year follow-up period among adults who had received GH treatment as children (N = 6928) for the diagnoses of idiopathic isolated GHD, neurosecretory dysfunction, idiopathic short stature or born SGA [52]. However, the preliminary data from Belgium, The Netherlands, and Sweden (N = 2543) contrasted with the report from France in that the majority of the 21 deaths that occurred over the follow-up period were due to accidents or suicide and not a single case of death was related to cancer or cardiovascular disease [53]. Questions have been raised about the SAGhE data from France due to methodological limitations of the study, including the lack of an ideal control group of untreated patients, and in August 2011, the FDA stated the evidence of increased risk of death is inconclusive [54,55].

Additionally, multiple reports have demonstrated GH is safe for use at currently recommended doses in short-statured children born SGA [17,37]. GH treatment has been shown safe across heterogeneous groups of children born SGA, including very young (ages 2–5) children born SGA, preterm (gestation ≤36 weeks) children born SGA, and in children born SGA who were both preterm and very young [46,56,57]. Continuous GH treatment over 6 years was well tolerated in 54 children with short stature born SGA [37]. Relative insulin resistance occurred but there was no adverse effect on glucose levels or development of diabetes and no GH related adverse events were detected [37]. Similarly, among 84 SGA children participating in the US SGA trial from the KIGS Database, a reduction in insulin sensitivity occurred during GH treatment, but no patients developed impaired glucose tolerance or overt diabetes mellitus [17].

The International SGA Advisory Board states that because insulin resistance may increase during GH therapy, reviewing for a family history of type 2 diabetes mellitus (T2DM) is important [1]. Annual screening of carbohydrate status, such as hemoglobin A1c, a fasting or postprandial glucose, and insulin levels, is suitable in lean children without a family history of diabetes. If pubertal children are obese, have a family history of T2DM, or have acanthosis nigricans, glucose homeostasis should be monitored more frequently and intensely (using A1c in addition to oral glucose tolerance test with insulin measurements when indicated). Additionally, fasting serum lipids and blood pressure should be periodically monitored during long-term GH therapy.

## Conclusions

SGA diagnosis is challenging, and guidelines for the selection of appropriate reference comparison data continue to evolve. Early diagnosis of SGA with failure to show catch-up growth and early referral to GH therapy are needed. In short-statured children born SGA without catch-up growth, early referral for GH evaluation and therapy is critical for optimal growth acceleration, normalization of prepubertal height, and improvements in AH. The average age of treatment referral varies, and often exceeds the International Societies of Pediatric Endocrinology and Growth Hormone Research Society consensus guidelines (that recommend referral at ages 2–4) by many years. The reasons for this referral delay are not known but likely involve parental and physician attitudes about the importance of early growth in children born SGA. Most children born SGA will do well developmentally, and it is essential that parents understand the benefits of GH treatment for short stature that accrue at a younger age of treatment initiation. Optimizing eventual height in short-statured patients born SGA without catch-up growth is most efficiently done by lowering the age of referral for GH evaluation and treatment to a time in childhood where initiation of treatment provides optimal benefit and aligns with consensus recommendations for SGA management.

## Abbreviations

AH, adult height; ANSWER, American Norditropin Studies: Web-Enabled Research; EMA, European Medicines Agency; GH, growth hormone; GHD, GH deficiency; HSDS, height standard deviation score; ΔHSDS, change in height standard deviation score; IGF-1, IGF-2, insulin-like growth factors; IOS, international outcome study; ISS, idiopathic short stature; IUGR, intrauterine growth retardation; KIGS, Pfizer International Growth Database; MPHD, multiple pituitary hormone deficiency; SD, standard deviation; SDS, SD score; SGA, small for gestational age; TS, Turner Syndrome; T2DM, type 2 diabetes mellitus.

## Competing interests

Dr. Houk has no competing interests to declare. Dr. Lee has served as an advisory board member for Novo Nordisk Inc., and his institution has received grant funding from Novo Nordisk Inc. as part of the American Norditropin® Studies Web-Enabled Research (ANSWER) Program. Dr. Lee is also a member of the Editorial Board for the *International Journal of Pediatric Endocrinology*.

## Authors’ contributions

CH & PL were the primary investigators, interpreted the data, critically reviewed and provided final approval of this manuscript.
